# More published full-time researchers, early career researchers, clinician-researchers and graduate students unite to call for actions against the pseudoscientific claim that chiropractic care boosts immunity

**DOI:** 10.1186/s12998-020-00334-5

**Published:** 2020-07-20

**Authors:** Pierre Côté, André Bussières, J. David Cassidy, Jan Hartvigsen, Greg N. Kawchuk, Charlotte Leboeuf-Yde, Silvano Mior, Michael Schneider

**Affiliations:** 1 Faculty of Health Sciences, Ontario Tech University, Oshawa, Canada; 2 Centre for Disability Prevention and Rehabilitation at Ontario Tech University and CMCC, Oshawa, Canada; 3 Division of Epidemiology, Dalla Lana School of Public Health, University of Toronto, Toronto, Canada; 4 Département chiropratique, Université du Québec à Trois-Rivières, Trois-Rivières, Canada; 5 School of Physical and Occupational Therapy, Faculty of Medicine McGill University, Montreal, Canada; 6 Department of Sports Science and Clinical Biomechanics, University of Southern Denmark, Odense, Denmark; 7 Nordic Institute of Chiropractic and Clinical Biomechanics, Odense, Denmark; 8 Faculty of Rehabilitation Medicine, University of Alberta, Edmonton, Canada; 9 Institute for Regional Health Research, University of Southern Denmark, Odense, Denmark; 10 Canadian Memorial Chiropractic College, Toronto, Canada; 11 School of Health and Rehabilitation Sciences, University of Pittsburgh, Pittsburgh, USA; 12 Clinical and Translational Science Institute, University of Pittsburgh, Pittsburgh, USA

On May 4, 2020 we published a commentary entitled: *“A united statement of the global chiropractic research community against the pseudoscientific claim that chiropractic care boosts immunity”* [1]. Our paper generated significant interest within the research community and several individuals contacted us to ask whether they could co-sign the commentary. We believe that it is important for members of the research community to formally add their voice to this important public health discussion.

On May 6, 2020, the authors of the commentary were asked to inform their networks of the opportunity to add co-signatories. This included an invitation made on social media. Specifically, we invited full-time researchers, early career researchers, clinician-researchers, graduate students and individuals who have published in the peer-reviewed literature during the course of their career to add their name to the list of co-signatories [2].

On May 20, 2020, 71 new individuals had responded to our call. Of those, 52 are affiliated with academic or research institutions, 15 are in private practice, two have retired and one is currently inactive. Therefore, 224 signatories are now calling for “regulatory authorities and professional leaders to take robust political and regulatory action against those claiming that chiropractic adjustments have a clinical impact on the immune system.”

It is likely that other individuals in the research community would like to add their signatures to the commentary. Therefore, a webpage has been created (https://nikkb.dk/table/cmt-signatories/) to display the names of all signatories, and provide the opportunity to those who qualify (publication in the peer reviewed literature) to add their names to the list.

## Data Availability

Not applicable.

## References

[1] Côté P, Bussières A, Cassidy JD (2020). A united statement of the global chiropractic research community against the pseudoscientific claim that chiropractic care boosts immunity. Chiropr Man Therap.

[2] Kawchuck G. https://twitter.com/GNK1/status/1258000331704635394?s=20.

